# Supplementary material for: Amplicon sequencing provides more accurate microbiome information in healthy children compared to culturing
Source: Commun Biol. 2019 Aug 5;2:291. doi: 10.1038/s42003-019-0540-1 (PMC6683184; doi:10.1038/s42003-019-0540-1)
Supplement: Supplementary file 1 — Supplementary Information [file 42003_2019_540_MOESM1_ESM.pdf]

Supplementary Figures:

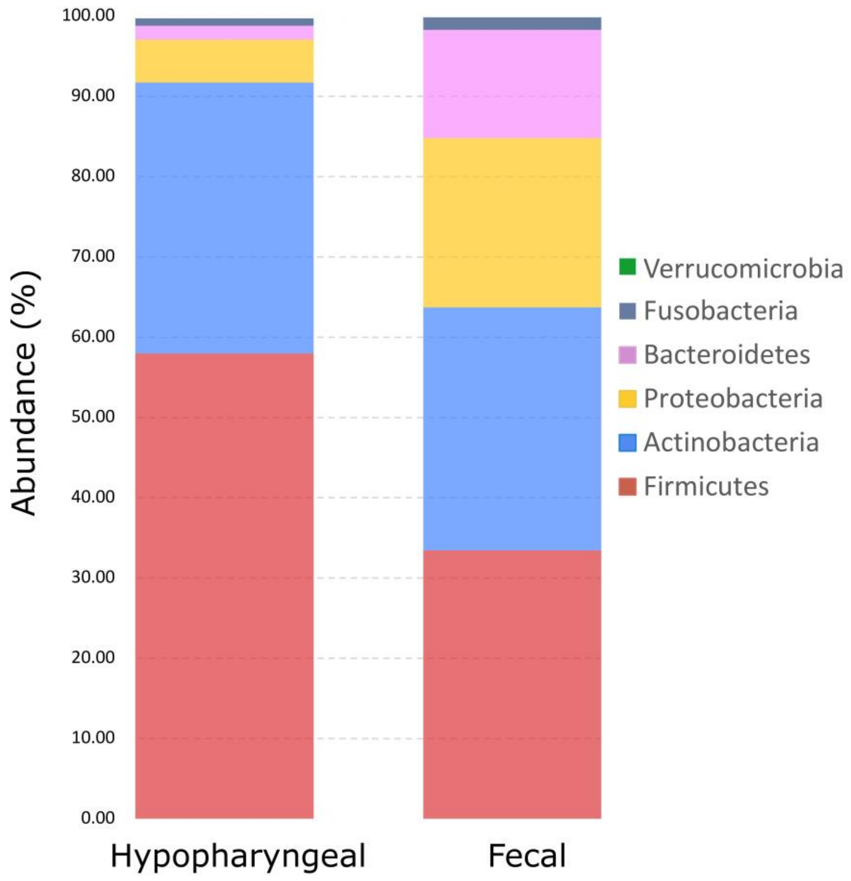

Supplementary Figure 1: Bar plot depicting the relative abundance (in %) of bacterial phylum distribution (top 5 phyla) among fecal, and hypopharyngeal samples using 16S rRNA gene sequences.

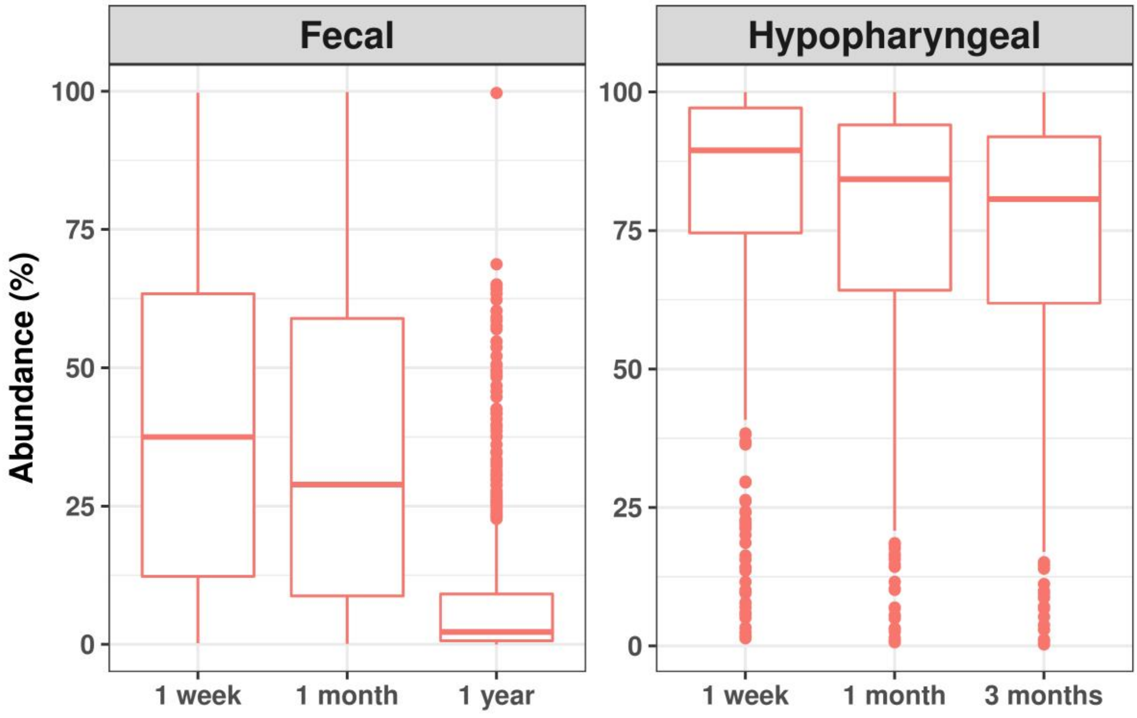

Supplementary Figure 2: Boxplot shows the bacterial species identified by sequencing and their abundance.

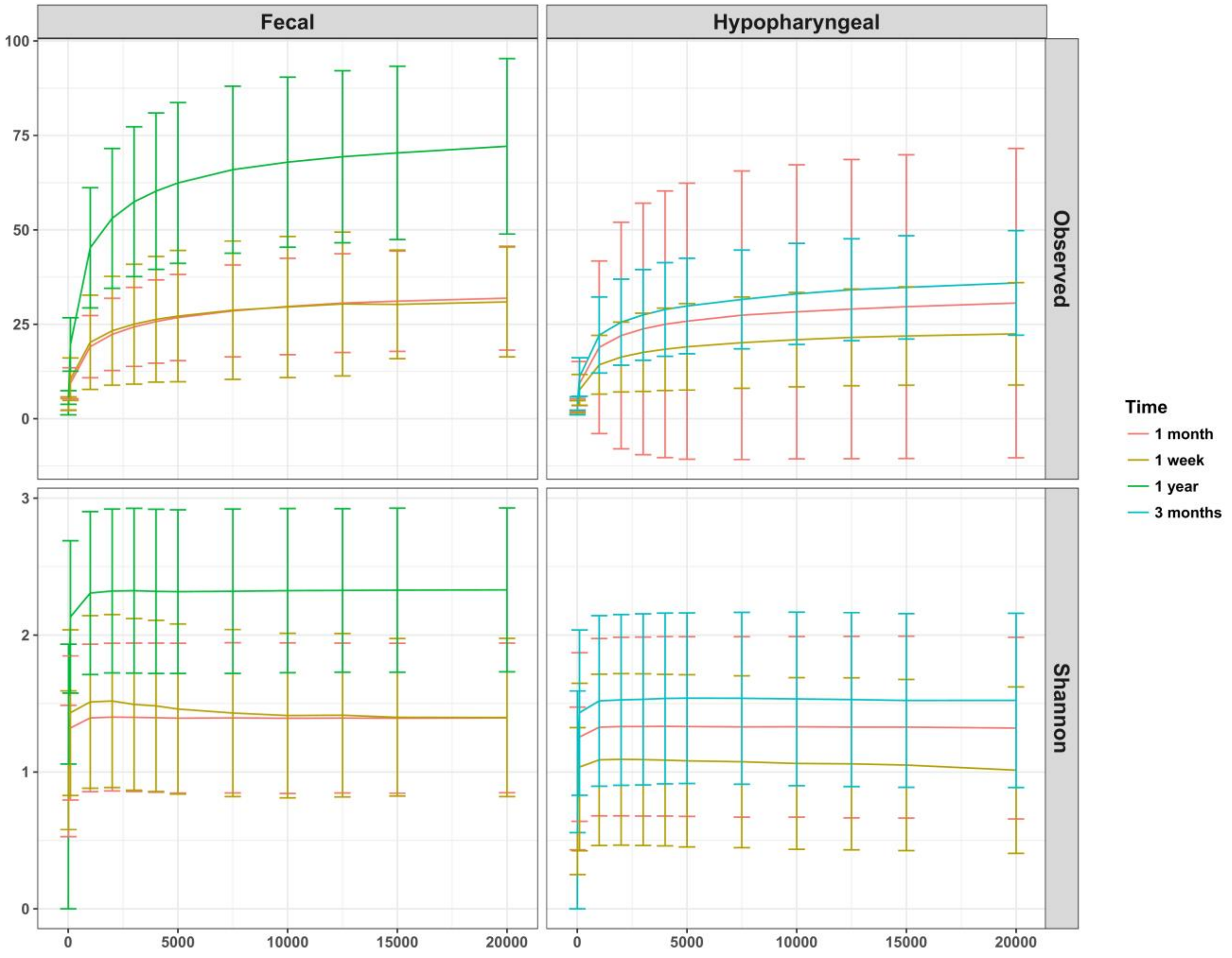

Supplementary Figure 3: Rarefaction curves of the fecal, and hypopharyngeal samples, among different time points generated from the 16S rRNA gene sequences. The rarefaction curves was calculated using richness and Shannon diversity index at increasing sequencing depth. Error bars represent standard deviation.

Supplementary tables:

Supplementary Table 1: List of cultured bacteria and the percentage of samples they were isolated from.

Species	Hypopharyngeal	Fecal
Staphylococcus aureus	48.28%	12.71%
Staphylococcus epidermidis	41.54%	29.46%
Corynebacterium sp	28.42%	2.95%
Moraxella catarrhalis	20.59%	0.00%
Streptococcus mitis/oralis	20.06%	0.72%
Staphylococcus hominis	10.14%	7.75%
Streptococcus pneumoniae	9.25%	0.05%
Micrococcus luteus	7.58%	2.89%
Haemophilus influenzae	5.90%	0.00%
Streptococcus salivarius	5.80%	1.65%
Streptococcus pluranimalium	5.80%	0.10%
Escherichia coli	5.43%	56.59%
Staphylococcus lugdunensis	5.02%	2.95%
Streptococcus agalactiae	4.55%	0.88%
Streptococcus sanguinis	3.61%	0.41%
Rothia mucilaginosa	3.34%	0.10%
Enterococcus faecalis	2.77%	23.20%
Gemella haemolysans	1.99%	0.00%
Staphylococcus intermedius	1.93%	0.10%
Micrococcus lylae	1.72%	0.41%
Kocuria kristinae	1.62%	0.10%
Staphylococcus haemolyticus	1.52%	1.55%
Klebsiella pneumoniae	1.46%	14.99%
Enterobacter cloacae	1.46%	7.60%
Sphingomonas paucimobilis	1.41%	0.31%
Streptococcus parasanguinis	1.36%	0.36%
Acinetobacter lwoffii	1.25%	0.26%
Haemophilus parainfluenzae	1.25%	0.00%
Streptococcus dysgalactiae	1.20%	0.10%
Granulicatella adiacens	1.15%	0.05%
Streptococcus vestibularis	1.10%	0.00%
Moraxella nonliquefaciens	1.10%	0.00%
Kocuria rosea	1.04%	0.00%
Staphylococcus pasteuri	0.94%	0.98%
Staphylococcus warneri	0.89%	1.86%
Staphylococcus capitis	0.89%	0.26%
Klebsiella oxytoca	0.84%	7.29%

Lactobacillus sp	0.84%	1.96%
Gemella sp	0.73%	0.10%
Stenotrophomonas maltophilia	0.68%	0.98%
Serratia marcescens	0.68%	0.36%
Streptococcus constellatus	0.63%	0.05%
Bacillus sp	0.57%	0.36%
Gemella sanguinis	0.57%	0.05%
Erysipelothrix rhusiopathiae	0.57%	0.00%
Granulicatella elegans	0.52%	0.00%
Streptococcus gordonii	0.52%	0.00%
Streptococcus gallolyticus	0.47%	0.36%
Streptococcus sp	0.47%	0.26%
Dermacoccus nishinomiya	0.47%	0.00%
Streptococcus anginosus	0.47%	0.00%
Streptococcus pyogenes	0.42%	0.05%
Staphylococcus cohnii	0.42%	0.00%
Neisseria flavescens	0.42%	0.00%
Neisseria sicca	0.42%	0.00%
Acinetobacter sp	0.37%	0.05%
Neisseria cinerea	0.37%	0.05%
Pantoea sp	0.31%	0.57%
Staphylococcus auricularis	0.31%	0.00%
Citrobacter freundii	0.26%	4.24%
Staphylococcus lentus	0.26%	0.62%
Gemella morbillorum	0.26%	0.05%
Enterobacter aerogenes	0.21%	1.14%
Citrobacter koseri	0.21%	0.98%
Staphylococcus saprophyticus	0.21%	0.10%
Aeromonas salmonicida	0.21%	0.05%
Leclercia adecarboxylata	0.21%	0.05%
Leuconostoc mesenteroides	0.21%	0.05%
Acinetobacter baumannii	0.21%	0.00%
Staphylococcus sp	0.21%	0.00%
Streptococcus intermedius	0.21%	0.00%
Enterococcus gallinarum	0.16%	3.36%
Pseudomonas aeruginosa	0.16%	0.31%
Streptococcus alactolyticus	0.16%	0.10%
Staphylococcus xylosus	0.16%	0.05%
Acinetobacter junii	0.16%	0.00%
Acinetobacter ursingii	0.16%	0.00%
Neisseria elongata	0.16%	0.00%
Pasteurella canis	0.16%	0.00%
Pseudomonas oryzihabitans	0.16%	0.00%

Streptococcus suis ii	0.16%	0.00%
Raoultella planticola	0.10%	0.47%
Staphylococcus simulans	0.10%	0.36%
Streptococcus uberis	0.10%	0.16%
Lactococcus raffinolactis	0.10%	0.10%
Kocuria varians	0.10%	0.05%
Gardnerella vaginalis	0.10%	0.00%
Staphylococcus caprae	0.10%	0.00%
Pseudomonas fluorescens	0.10%	0.00%
Staphylococcus chromogenes	0.10%	0.00%
Staphylococcus vitulinus	0.10%	0.00%
Granulicatella sp	0.10%	0.00%
Neisseria perflava	0.10%	0.00%
Pseudomonas luteola	0.10%	0.00%
Streptococcus equinus	0.10%	0.00%
Streptococcus thermophilus	0.10%	0.00%
Enterococcus faecium	0.05%	2.33%
Citrobacter amalonaticus	0.05%	1.91%
Citrobacter braakii	0.05%	1.29%
Enterobacter sp	0.05%	0.67%
Streptococcus infantarius	0.05%	0.62%
Lactococcus garvieae	0.05%	0.26%
Pediococcus pentosaceus	0.05%	0.21%
Aerococcus viridans	0.05%	0.10%
Staphylococcus sciuri	0.05%	0.05%
Alloiococcus otitis	0.05%	0.05%
Escherichia vulneris	0.05%	0.05%
Oligella ureolytica	0.05%	0.00%
Streptococcus ovis	0.05%	0.00%
Actinobacillus ureae	0.05%	0.00%
Comamonas testosteroni	0.05%	0.00%
Haemophilus haemolyticus	0.05%	0.00%
Neisseria meningitidis	0.05%	0.00%
Neisseria mucosa	0.05%	0.00%
Neisseria sp	0.05%	0.00%
Neisseria subflava	0.05%	0.00%
Pasteurella pneumotropica	0.05%	0.00%
Pseudomonas pseudoalcaligene	0.05%	0.00%
Pseudomonas stutzeri	0.05%	0.00%
Streptococcus porcinus	0.05%	0.00%
Clostridium XI difficile	0.00%	8.68%
Enterococcus avium	0.00%	6.67%
Clostridium sp	0.00%	5.99%

Citrobacter farmeri	0.00%	2.17%
Proteus mirabilis	0.00%	1.76%
Enterococcus casseliflavus	0.00%	1.29%
Escherichia hermannii	0.00%	0.83%
Proteus penneri	0.00%	0.57%
Citrobacter youngae	0.00%	0.52%
Enterococcus durans	0.00%	0.52%
Proteus sp	0.00%	0.47%
Proteus vulgaris	0.00%	0.47%
Enterococcus raffinosus	0.00%	0.41%
Morganella morganii	0.00%	0.36%
Serratia liquefaciens	0.00%	0.36%
Lactococcus lactis	0.00%	0.31%
Enterococcus sp	0.00%	0.31%
Serratia fonticola	0.00%	0.31%
Raoultella ornithinolytica	0.00%	0.26%
Enterobacter amnigenus	0.00%	0.21%
Enterobacter sakazakii	0.00%	0.21%
Hafnia alvei	0.00%	0.21%
Streptococcus thoraltensis	0.00%	0.10%
Citrobacter sp	0.00%	0.10%
Clostridium sensu stricto perfringens	0.00%	0.10%
Enterobacter aburiae	0.00%	0.10%
Enterococcus hirae	0.00%	0.10%
Escherichia sp	0.00%	0.10%
Lactococcus sp	0.00%	0.10%
Providencia rettgeri	0.00%	0.10%
Pantoea agglomerans	0.00%	0.05%
Acinetobacter haemolyticus	0.00%	0.05%
Aeromonas caviae	0.00%	0.05%
Aeromonas sobria	0.00%	0.05%
Aeromonas veronii	0.00%	0.05%
Enterococcus columbae	0.00%	0.05%
Klebsiella sp	0.00%	0.05%
Kluyvera cryocrescens	0.00%	0.05%
Kluyvera intermedia	0.00%	0.05%
Leuconostoc mesenteroides ss	0.00%	0.05%
Listeria sp	0.00%	0.05%
Oligella urethralis	0.00%	0.05%
Pasteurella aerogenes	0.00%	0.05%
Providencia alcalifaciens	0.00%	0.05%
Salmonella sp	0.00%	0.05%
Shigella sonnei	0.00%	0.05%

Streptococcus bovis	0.00%	0.05%
Yersinia enterocolitica	0.00%	0.05%
Moraxella sp	0.00%	0.00%
Aerococcus urinae	0.00%	0.00%
Staphylococcus carnosus	0.00%	0.00%
Globicatella sanguinis	0.00%	0.00%
Myroides sp	0.00%	0.00%
Streptococcus mutans	0.00%	0.00%
Rhizobium radiobacter	0.00%	0.00%
Facklamia hominis	0.00%	0.00%
Kocuria sp	0.00%	0.00%
Rothia dentocariosa	0.00%	0.00%
Shigella sp	0.00%	0.00%

Supplementary Table 2: List of bacteria at the phylum level and their percentage observed by amplicon sequencing.

**Top Phyla**	**Hypopharyngeal**		**Fecal**	
1	Firmicutes	61.90	Bacteroidetes	34.27
2	Proteobacteria	29.78	Firmicutes	26.59
3	Actinobacteria	5.61	Proteobacteria	21.69
4	Bacteroidetes	1.63	Actinobacteria	15.89
5	Fusobacteria	0.69	Verrucomicrobia	1.45

Supplementary Table 3: List of bacteria at the family level and their percentage observed by amplicon sequencing.

**Family**	**Fecal**	**Hypopharyngeal**
Bacteroidaceae	29.10746	0.083984484
Enterobacteriaceae	23.85815	2.853771318
Bifidobacteriaceae	15.29383	0.078261794
Lachnospiraceae	4.620658	0.232434385
Veillonellaceae	4.083705	2.797286268
Ruminococcaceae	3.406203	0.112350498
Clostridiaceae_1	3.157983	0.207471041
Porphyromonadaceae	2.475381	0.196489668
Streptococcaceae	2.309014	25.89503045
Pasteurellaceae	1.905041	6.157531999
Prevotellaceae	1.810898	0.921787929
Staphylococcaceae	1.807857	26.39256478
Verrucomicrobiaceae	1.449054	0.001484809
Rikenellaceae	0.857142	0.000866138
Enterococcaceae	0.800218	0.146686711
Lactobacillaceae	0.738956	0.796197674
Alcaligenaceae	0.436442	0.00757871
Coriobacteriaceae	0.270203	0.037151146
Peptostreptococcaceae	0.254111	0.010702996
Actinomycetaceae	0.229308	0.178950365
Erysipelotrichaceae	0.215529	0.014074745
Acidaminococcaceae	0.191138	9.28006E-05
Neisseriaceae	0.111629	2.491199419
Moraxellaceae	0.095474	15.8482278
Desulfovibrionaceae	0.080491	0.001113606
Fusobacteriaceae	0.066521	0.451103378
Micrococcaceae	0.053565	0.999276157
XI_Clostridiales	0.03513	0.105730742
Corynebacteriaceae	0.03041	4.185242243
Xanthomonadaceae	0.030093	0.050885626
Anaeroplasmataceae	0.021445	0.001268274
Helicobacteraceae	0.018499	0.167319363
Christensenellaceae	0.017581	0.001577609
Bacteroidales_S24_7_group	0.017105	3.09335E-05
Rhodospirillaceae	0.013748	0.002598415
Leuconostocaceae	0.012227	0.009280055
XI_Bacillales	0.011435	3.307132647
Pseudomonadaceae	0.008299	0.408693554

Bacillaceae	0.007697	0.145975241
Rhodobacteraceae	0.007476	0.142139485
Order_Gastranaerophilales	0.006557	0.000433069
Campylobacteraceae	0.00643	0.045472265
Listeriaceae	0.006145	0.110277968
Rhizobiaceae	0.005195	0.002258146
Eubacteriaceae	0.004181	0
XIII_Clostridiales	0.004023	0.000587737
Dermabacteraceae	0.004023	0.008599516
Victivallaceae	0.003991	0
Carnobacteriaceae	0.003389	1.548593453
Flavobacteriaceae	0.003358	0.219999134
Clostridiales_grp_vadinBB60	0.002502	0.007857112
Deinococcaceae	0.002217	0.084757822
Chitinophagaceae	0.002091	0.148759257
Comamonadaceae	0.001489	0.838143494
Phyllobacteriaceae	0.001489	0.001206407
Sphingomonadaceae	0.001489	0.37667737
Microbacteriaceae	0.001425	0.015126487
Roseiarcaceae	0.001235	9.28006E-05
Thermaceae	0.001204	0.000494936
Solibacteraceae_Subgroup_3	0.001077	0.012899274
Methylobacteriaceae	0.001077	0.027004956
Acetobacteraceae	0.001045	0.00176321
Colwelliaceae	0.000887	0
Caulobacteraceae	0.00076	0.069724135
Anaplasmataceae	0.000697	3.09335E-05
Intrasporangiaceae	0.000665	0.007764311
Order_Rhizobiales	0.000665	0.000123734
Class_Mollicutes	0.000539	0.000587737
Paenibacillaceae	0.000539	0.006217636
Cytophagaceae	0.000507	0.005660833
Polyangiaceae	0.000475	0.003402686
Peptococcaceae	0.000412	0.00176321
Order_Obscuribacterales	0.00038	0.062114491
ODP1230B823	0.00038	0.007393109
Oceanospirillaceae	0.000348	0
Succinivibrionaceae	0.000317	0.01787957
Solirubrobacterales_Elev_16S_1332	0.000317	0.006093902
Xanthobacteraceae	0.000317	0.003340819
Aeromonadaceae	0.000317	0.000278402
Acidothermaceae	0.000317	0
Order_Lactobacillales	0.000285	3.09335E-05

Acidobacteriaceae_subgrp_1	0.000285	0.003495487
Flavobacteriales_NS9	0.000285	0
Syntrophorhabdaceae	0.000285	0
Dermacoccaceae	0.000222	0.016642229
XII_Bacillales	0.000222	0.000835205
Frankiaceae	0.000222	0.000958939
Sporichthyaceae	0.000222	0
Order_Clostridiales	0.00019	3.09335E-05
Bradyrhizobiaceae	0.00019	0.01506462
Nitrosomonadaceae	0.00019	0.001361075
Spirochaetaceae	0.00019	0.00114454
Conexibacteraceae	0.00019	0
Leptotrichiaceae	0.000158	0.23961098
Burkholderiaceae	0.000158	0.00863045
Phylum_Saccharibacteria	0.000158	0.001701343
Phylum_Firmicutes	0.000158	0.000340269
Brevibacteriaceae	0.000127	0.02202466
Xanthomonadales_Incertae_Sedis	0.000127	0.019333445
Oligoflexales_0319_6G20	0.000127	0.003248019
Order_Acidimicrobiales	0.000127	0.001392008
Cellulomonadaceae	0.000127	0.00061867
Alteromonadaceae	0.000127	0.000433069
Class_Acidobacteria_subgrp_13	9.5E-05	3.09335E-05
Hydrogenophilaceae	9.5E-05	0.003464553
Hyphomicrobiaceae	9.5E-05	0.111762777
Planctomycetaceae	9.5E-05	0.005537099
Sphingobacteriaceae	9.5E-05	0.00977499
Planococcaceae	9.5E-05	0.008259248
Nocardioidaceae	9.5E-05	0.00405229
Rhodocyclaceae	9.5E-05	0.002103479
Nocardiaceae	9.5E-05	0.001732277
Class_Verrucomicrobia_OPB35	9.5E-05	0.001515742
Order_Betaproteobacteria_SC_I_84	9.5E-05	0.001330141
Coxiellaceae	9.5E-05	0.000494936
Solirubrobacterales_YNPFFP1	9.5E-05	0.000278402
Sphingobacteriales_KD3_93	9.5E-05	6.1867E-05
Class_Chloroflexi_JG37_AG_4	9.5E-05	0
FamilyI	8.45E-05	0.00087645
Synergistaceae	6.34E-05	0
Acidimicrobiaceae	6.34E-05	0.001020806
Brucellaceae	6.34E-05	0.000123734
Order_Thermomicrobia_JG30_KF_CM45	6.34E-05	0.019178777
Order_Myxococcales	6.34E-05	0.019147844

Vibrionaceae	6.34E-05	0.012373405
Erythrobacteraceae	6.34E-05	0.000866138
Tepidisphaeraceae	6.34E-05	0.000742404
Pseudoalteromonadaceae	6.34E-05	0.000309335
Propionibacteriaceae	6.34E-05	0.000216535
Methylophilaceae	6.34E-05	9.28006E-05
Myxococcales_mle1_27	6.34E-05	6.1867E-05
Holophagaceae	6.34E-05	0
Ktedonobacterales_1959_1	6.34E-05	0
Thermosporotrichaceae	6.34E-05	0
Victivallales_vadinBE97	6.34E-05	0
Xiphinematobacteraceae	6.34E-05	0
Defluviitaleaceae	6.34E-05	3.09335E-05
Aerococcaceae	3.17E-05	0.0057227
Oxalobacteraceae	3.17E-05	0.008846984
Mycobacteriaceae	3.17E-05	0.016178227
Order_Bacteroidales	3.17E-05	3.09335E-05
Rhodospirillales_DA111	3.17E-05	0.001639476
Alicyclobacillaceae	3.17E-05	0.021220389
Gemmatimonadaceae	3.17E-05	0.00924912
Order_Bacillales	3.17E-05	0.005042162
Order_Micrococcales	3.17E-05	0.003712021
Anaerolineaceae	3.17E-05	0.00352642
Crenotrichaceae	3.17E-05	0.003248019
Desulfurellaceae	3.17E-05	0.002876817
Phylum_Proteobacteria	3.17E-05	0.001732277
Order_Ktedonobacterales	3.17E-05	0.001546676
Archangiaceae	3.17E-05	0.001020806
Bacteroidales_RF16_group	3.17E-05	0.000835205
envOPS_17	3.17E-05	0.000773338
Solirubrobacterales_TM146	3.17E-05	0.000494936
Pseudonocardiaceae	3.17E-05	0.000402136
Shewanellaceae	3.17E-05	0.000278402
Class_Acidobacteria_subgrp_2	3.17E-05	0.000154668
Solirubrobacteraceae	3.17E-05	0.000123734
Order_Xanthomonadales	3.17E-05	9.28005E-05
Gsoil_1167	3.17E-05	6.1867E-05
Rhizobiales_MNG7	3.17E-05	3.09335E-05
BacC_u_018	3.17E-05	0
Brevinemataceae	3.17E-05	0
Caldicoprobacteraceae	3.17E-05	0
Chthonomonadaceae	3.17E-05	0
Demequinaceae	3.17E-05	0

Fibrobacterales_B122	3.17E-05	0
Fimbriimonadaceae	3.17E-05	0
Ktedonobacteraceae	3.17E-05	0
LD12_freshwater_group	3.17E-05	0
Order_Actinobacteria_PeM15	3.17E-05	0
Order_Solirubrobacterales	3.17E-05	0
Sphingobacteriales_S15A_MN91	3.17E-05	0
XII_Clostridiales	3.17E-05	0
Ambiguous_taxa	1.06E-05	4.12447E-05
Mycoplasmataceae	0	0.055834994
Entomoplasmataceae	0	0
Class_Acidobacteria_subgrp_6	0	0.024808676
Order_Ardenticatenales	0	0
Phylum_TM6_Dependentiae	0	0.003217085
Chthoniobacteraceae	0	0.001886944
Brocadiaceae	0	0.000309335
Spiroplasmataceae	0	0
Nitrospirales_0319_6A21	0	0.005351498
Class_Actinobacteria	0	0
Order_Acholeplasmatales	0	0
Filobacteriaceae	0	0.033036988
Blastocatellaceae_subgrp_4	0	0.019147846
Class_Chloroflexi_KD4_96	0	0.018838511
Phaselicystidaceae	0	0.010455526
Class_Acidobacteria_subgrp_17	0	0.00900165
Dietziaceae	0	0.005598965
Sandaracinaceae	0	0.005475231
Class_Chloroflexi_S085	0	0.003773888
Order_Holophagae_subgrp_7	0	0.003712022
Caldilineaceae	0	0.003588287
Trueperaceae	0	0.003278952
Class_Chloroflexi_TK10	0	0.003217085
X_Bacillales	0	0.00300055
Rhizobiales_C2U	0	0.002969617
Cardiobacteriaceae	0	0.00281495
Rhizobiales_Incertae_Sedis	0	0.002629348
Streptomycetaceae	0	0.002567482
Order_Gaiellales	0	0.002505615
Order_Frankiales	0	0.002412814
Micromonosporaceae	0	0.002350947
Acidimicrobiales_Incertae_Sedis	0	0.002196279
Geodermatophilaceae	0	0.002196279
Nakamurellaceae	0	0.002134413

Iamiaceae	0	0.002103479
Syntrophaceae	0	0.002041612
Rhizobiales_1174_901_12	0	0.001917878
Hyphomonadaceae	0	0.001639476
Order_Gammaproteobacteria_E01_9C_26	0	0.001639476
Phylum_SR1_Absconditabacteria	0	0.001422941
Streptosporangiaceae	0	0.001392008
Haliangiaceae	0	0.001330141
Class_Chloroflexi_Gitt_GS_136	0	0.001175473
Rhodospirillales_Incertae_Sedis	0	0.00114454
Verrucomicrobiales_DEV007	0	0.001082673
Chtoniobacterales_DA101	0	0.001020806
Class_Gammaproteobacteria	0	0.001020806
Order_Sphingobacteriales	0	0.001020806
Myxococcales_BIrii41	0	0.000958939
Phylum_Hydrogenedentes	0	0.000958939
Phycisphaeraceae	0	0.000928006
Class_Cloacimonetes_W5	0	0.000897072
Pylum_Verrucomicrobia	0	0.000897072
Order_Ktedonobacteria_C0119	0	0.000866138
Class_Clostridia	0	0.000835205
Phylum_Tectomicrobia	0	0.000835205
Halomonadaceae	0	0.000680537
Class_Alphaproteobacteria	0	0.000649604
Gaiellaceae	0	0.000649604
Halieaceae	0	0.000649604
Roseiflexaceae	0	0.00061867
Bacteroidales_grp_BS11	0	0.000587737
Phylum_Armatimonadetes	0	0.000587737
Kineosporiaceae	0	0.000556803
Flammeovirgaceae	0	0.00052587
Promicromonosporaceae	0	0.00052587
Saprospiraceae	0	0.000464003
Nitrospiraceae	0	0.000433069
Opitutaceae	0	0.000433069
Class_Candidatus_Nomurabacteria	0	0.000402136
Order_Phycisphaerae_CPla_3	0	0.000402136
Rhizobiales_KF_JG30_B3	0	0.000402136
Nocardiopsaceae	0	0.000371202
Ignavibacteriaceae	0	0.000340269
Methylococcaceae	0	0.000340269
Fibrobacteraceae	0	0.000309335
Class_Chloroflexi_JG30_KF_CM66	0	0.000278402

Class_Acidobacteria_subgrp_15	0	0.000247468
Class_Bacteroidetes_WCHB1_32	0	0.000247468
Holophagae_subgrp_10_ABS_19	0	0.000247468
Patulibacteraceae	0	0.000247468
Pylum_Chloroflexi	0	0.000216535
Rhodospirillales_KCM_B_60	0	0.000216535
X.Caedibacter._caryophilus_group	0	0.000185601
Class_Acidobacteria_subgrp_12	0	0.000185601
Cytophagales_MWH_CFBk5	0	0.000185601
III_Thermoanaerobacterales	0	0.000185601
Aurantimonadaceae	0	0.000154668
Bogoriellaceae	0	0.000154668
Euzebyaceae	0	0.000154668
Piscirickettsiaceae	0	0.000154668
Bdellovibrionaceae	0	0.000123734
Class_Bacteroidetes_VC21_Bac22	0	0.000123734
Halanaerobiaceae	0	0.000123734
Legionellaceae	0	0.000123734
Oligoflexales_053A03_B_DI_P58	0	0.000123734
Order_TRA3_20	0	0.000123734
Parachlamydiaceae	0	0.000123734
Phylum_Cyanobacteria	0	0.000123734
Rhodobiaceae	0	0.000123734
Sphingobacteriales_PHOS_HE51	0	0.000123734
Thermoactinomycetaceae	0	0.000123734
Thermomonosporaceae	0	0.000123734
Bartonellaceae	0	9.28006E-05
Chthoniobacterales_01D2Z36	0	9.28006E-05
Class_Cyanobacteria_ML635J_21	0	9.28006E-05
FamilyII	0	9.28006E-05
Order_Rhodospirillales	0	9.28006E-05
Order_Vampirovibrionales	0	9.28006E-05
Rubrobacteriaceae	0	9.28006E-05
Beijerinckiaceae	0	6.1867E-05
Cellvibrionaceae	0	6.1867E-05
Class_Acidobacteria_subgrp_11	0	6.1867E-05
Class_Actinobacteria_MB_A2_108	0	6.1867E-05
Class_Candidatus_Azambacteria	0	6.1867E-05
Class_Verrucomicrobia_S_BQ2_57	0	6.1867E-05
Desulfobacteraceae	0	6.1867E-05
Ignavibacteriales_BSV26	0	6.1867E-05
Order_Sphingomonadales	0	6.1867E-05
Rhizobiales_JG34_KF_361	0	6.1867E-05

Rhodospirillales_I_10	0	6.1867E-05
Rickettsiales_Incertae_Sedis	0	6.1867E-05
Aquificaceae	0	3.09335E-05
Chlorobiales_OPB56	0	3.09335E-05
Chromatiaceae	0	3.09335E-05
Class_Thermomicrobia	0	3.09335E-05
Clostridiales_Incertae_Sedis	0	3.09335E-05
Desulfohalobiaceae	0	3.09335E-05
Geobacteraceae	0	3.09335E-05
Kallotenuales_AKIW781	0	3.09335E-05
Myxococcales_P3OB_42	0	3.09335E-05
Order_Alphaproteobacteria_E6aD10	0	3.09335E-05
Order_Armatimonadales	0	3.09335E-05
Order_Bradymonadales	0	3.09335E-05
Order_Deltaproteobacteria_NB1_j	0	3.09335E-05
Order_Rickettsiales	0	3.09335E-05
Phylum_Gracilibacteria	0	3.09335E-05
Phylum_Microgenomates	0	3.09335E-05
Rhizobiales_alphaI_cluster	0	3.09335E-05
Rhizobiales_P_102	0	3.09335E-05
Rhodospirillales_JG37_AG_20	0	3.09335E-05
Sphingobacteriales_NS11_12	0	3.09335E-05

Supplementary Table 4: Complete list of species identified by culturing and their percentage and which unique bacterial species they belong to.

Species identified by Culturing			
**Genus**	**Species**	**Percent**	**Unique bacterial species group**
Acinetobacter	baumannii	0.0331	Acinetobacter_baumannii
Acinetobacter	haemolyticus	0.011	Acinetobacter_haemolyticus
Acinetobacter	junii	0.0221	Acinetobacter_junii
Acinetobacter	lwoffii	0.2978	Acinetobacter_lwoffii
Acinetobacter	ursingii	0.0331	Acinetobacter_ursingii
Aerococcus	viridans	0.0331	Aerococcus_viridans
Aeromonas	salmonicida	0.0552	Aeromonas_caviae_salmonicida_veronii
Aeromonas	caviae	0.011	Aeromonas_caviae_salmonicida_veronii
Aeromonas	sobria	0.011	Aeromonas_sobria
Alloiococcus	otitis	0.0221	Alloiococcus_otitis
Citrobacter	koseri	0.2316	Citrobacter_koseri
Clostridium_XI	difficile	1.6987	Clostridium_difficile
Clostridium_sensu_stricto	perfringens	0.0221	Clostridium_perfringens
Enterobacter	sakazakii	0.0441	Cronobacter_sakazakii
Dermacoccus	nishinomiya	0.0993	Dermacoccus_nishinomiyaensis
Enterobacter	aburiae	0.0221	Enterobacter_aburiae
Enterobacter	aerogenes	0.2096	Enterobacter_aerogenes
Enterobacter	amnigenus	0.0331	Enterobacter_amnigenus
Klebsiella	pneumoniae	3.2208	Enterobacteriaceae_GroupA
Enterobacter	cloacae	1.7759	Enterobacteriaceae_GroupA
Klebsiella	oxytoca	1.5442	Enterobacteriaceae_GroupA
Citrobacter	freundii	0.8824	Enterobacteriaceae_GroupA
Citrobacter	farmeri	0.4522	Enterobacteriaceae_GroupA
Citrobacter	amalonaticus	0.375	Enterobacteriaceae_GroupA
Citrobacter	braakii	0.2537	Enterobacteriaceae_GroupA
Citrobacter	youngae	0.0993	Enterobacteriaceae_GroupA
Leclercia	adecarboxylata	0.0552	Enterobacteriaceae_GroupA
Kluyvera	cryocrescens	0.011	Enterobacteriaceae_GroupA
Kluyvera	intermedia	0.011	Enterobacteriaceae_GroupA
Escherichia	vulneris	0.011	Enterobacteriaceae_GroupA
Enterococcus	casseliflavus	0.2537	Enterococcus_casseliflavus
Enterococcus	gallinarum	0.6949	Enterococcus_casseliflavus
Enterococcus	columbae	0.011	Enterococcus_columbae
Enterococcus	faecalis	4.9746	Enterococcus_faecalis_durans_hirae
Enterococcus	durans	0.1103	Enterococcus_faecalis_durans_hirae
Enterococcus	hirae	0.011	Enterococcus_faecalis_durans_hirae
Enterococcus	faecium	0.4743	Enterococcus_faecium

Enterococcus	avium	1.3788	Enterococcus_raffinosus_avium
Enterococcus	raffinosus	0.0772	Enterococcus_raffinosus_avium
Erysipelothrix	rhusiopathiae	0.0882	Erysipelothrix_rhusiopathiae
Escherichia	coli	12.2656	Escherichia/Shigella_coli_sonnei
Shigella	sonnei	0.011	Escherichia/Shigella_coli_sonnei
Escherichia	hermannii	0.1765	Escherichia_hermannii
Corynebacterium	sp	6.2321	excluded, only genus
Clostridium	sp	1.1802	excluded, only genus
Lactobacillus	sp	0.4853	excluded, only genus
Bacillus	sp	0.1985	excluded, only genus
Pantoea	sp	0.1875	excluded, only genus
Gemella	sp	0.1765	excluded, only genus
Enterobacter	sp	0.1544	excluded, only genus
Streptococcus	sp	0.1544	excluded, only genus
Proteus	sp	0.0993	excluded, only genus
Acinetobacter	sp	0.0882	excluded, only genus
Enterococcus	sp	0.0662	excluded, only genus
Staphylococcus	sp	0.0441	excluded, only genus
Granulicatella	sp	0.0221	excluded, only genus
Lactococcus	sp	0.0221	excluded, only genus
Citrobacter	sp	0.011	excluded, only genus
Escherichia	sp	0.011	excluded, only genus
Klebsiella	sp	0.011	excluded, only genus
Listeria	sp	0.011	excluded, only genus
Neisseria	sp	0.011	excluded, only genus
Salmonella	sp	0.011	excluded, only genus
Leuconostoc	sp	0.0552	excluded, only genus
Leuconostoc	sp	0.011	excluded, only genus
Gardnerella	vaginalis	0.0221	Gardnerella_vaginalis
Gemella	haemolysans	0.4081	Gemella_GroupA
Gemella	sanguinis	0.1213	Gemella_GroupA
Gemella	morbillorum	0.0552	Gemella_GroupA
Granulicatella	adiacens	0.2537	Granulicatella_adiacens
Granulicatella	elegans	0.0882	Granulicatella_elegans
Haemophilus	haemolyticus	0.011	Haemophilus_haemolyticus
Haemophilus	influenzae	1.2133	Haemophilus_influenzae
Haemophilus	parainfluenzae	0.2427	Haemophilus_parainfluenzae
Hafnia	alvei	0.0441	Hafnia_alvei
Kocuria	kristinae	0.353	Kocuria_kristinae
Kocuria	rosea	0.2206	Kocuria_rosea
Kocuria	varians	0.0221	Kocuria_varians
Lactococcus	garvieae	0.0441	Lactococcus_garvieae
Lactobacillus	garvieae	0.011	Lactococcus_garvieae

Lactococcus	lactis	0.0662	Lactococcus_lactis
Lactococcus	raffinolactis	0.0441	Lactococcus_raffinolactis
Micrococcus	luteus	1.9193	Micrococcus_luteus
Micrococcus	lylae	0.3971	Micrococcus_lylae
Moraxella	catarrhalis	4.2797	Moraxella_catarrhalis_nonliquefaciens
Moraxella	nonliquefaciens	0.2206	Moraxella_catarrhalis_nonliquefaciens
Morganella	morganii	0.0662	Morganella_morganii
Neisseria	cinerea	0.0882	Neisseria_cinerea_subflava_perflava
Neisseria	perflava	0.0221	Neisseria_cinerea_subflava_perflava
Neisseria	subflava	0.011	Neisseria_cinerea_subflava_perflava
Neisseria	elongata	0.0331	Neisseria_elongata
Neisseria	flavescens	0.0882	Neisseria_flavescens
Neisseria	meningitidis	0.011	Neisseria_meningitidis
Neisseria	sicca	0.0882	Neisseria_sicca_mucosa
Neisseria	mucosa	0.011	Neisseria_sicca_mucosa
Oligella	ureolytica	0.011	Oligella_ureolytica
Oligella	urethralis	0.011	Oligella_urethralis
Pantoea	agglomerans	0.011	Pantoea_agglomerans
Pasteurella	canis	0.0331	Pasteurella_canis
Pasteurella	pneumotropica	0.011	Pasteurella_pneumotropica
Pediococcus	pentosaceus	0.0441	Pediococcus_pentosaceus
Proteus	mirabilis	0.364	Proteus_mirabilis
Proteus	penneri	0.1103	Proteus_penneri
Proteus	vulgaris	0.0993	Proteus_vulgaris
Providencia	alcalifaciens	0.011	Providencia_alcalifaciens
Providencia	rettgeri	0.0221	Providencia_rettgeri
Pseudomonas	aeruginosa	0.0882	Pseudomonas_aeruginosa
Pseudomonas	pseudoalcaligene	0.011	Pseudomonas_aeruginosa
Pseudomonas	fluorescens	0.0221	Pseudomonas_fluorescens
Pseudomonas	luteola	0.0221	Pseudomonas_luteola
Pseudomonas	oryzihabitans	0.0331	Pseudomonas_oryzihabitans
Pseudomonas	stutzeri	0.011	Pseudomonas_stutzeri
Raoultella	ornithinolytica	0.0552	Raoultella_ornithinolytica
Raoultella	planticola	0.1213	Raoultella_planticola
Rothia	mucilaginosa	0.7059	Rothia_mucilaginosa
Serratia	fonticola	0.0552	Serratia_fonticola
Serratia	liquefaciens	0.0662	Serratia_liquefaciens
Serratia	marcescens	0.1655	Serratia_marcescens
Sphingomonas	paucimobilis	0.3419	Sphingomonas_paucimobilis
Staphylococcus	auricularis	0.0552	Staphylococcus_auricularis
Staphylococcus	simulans	0.0993	Staphylococcus_carnosus_simulans
Staphylococcus	chromogenes	0.0221	Staphylococcus_chromogenes
Staphylococcus	coctunansis	0.011	Staphylococcus_coctunansis

Staphylococcus	cohnii	0.0772	Staphylococcus_cohnii
Staphylococcus	lugdunensis	1.5222	Staphylococcus_GroupA
Streptococcus	lugdunensis	0.011	Staphylococcus_GroupA
Staphylococcus	epidermidis	13.4458	Staphylococcus_GroupA
Staphylococcus	aureus_capitis	12.045	Staphylococcus_GroupA
Staphylococcus	hominis	3.3752	Staphylococcus_GroupA
Staphylococcus	warneri	0.5184	Staphylococcus_GroupA
Staphylococcus	pasteuri	0.3309	Staphylococcus_GroupA
Staphylococcus	caprae	0.0221	Staphylococcus_GroupA
Streptococcus	ovis	0.011	Staphylococcus_GroupA
Staphylococcus	haemolyticus	0.5846	Staphylococcus_haemolyticus
Staphylococcus	intermedius	0.4081	Staphylococcus_intermedius
Staphylococcus	lentus	0.1765	Staphylococcus_lentus
Staphylococcus	saprophyticus	0.0552	Staphylococcus_saprophyticus
Staphylococcus	sciuri	0.011	Staphylococcus_sciuri
Staphylococcus	vitulinus	0.011	Staphylococcus_vitulinus
Staphylococcus	xylosus	0.0331	Staphylococcus_xylosus
Stenotrophomonas	maltophilia	0.3419	Stenotrophomonas_maltophilia
Streptococcus	agal_dys	0.0552	Streptococcus_agal_dys
Streptococcus	agalactiae	1.092	Streptococcus_agal_dys
Streptococcus	anginosus	0.0772	Streptococcus_anginosus
Streptococcus	constellatus	0.1434	Streptococcus_constellatus
Streptococcus	dysgalactiae	0.2758	Streptococcus_dysgalactiae
Streptococcus	infantarius	0.1434	Streptococcus_equinus_infantarius
Streptococcus	equinus	0.0221	Streptococcus_equinus_infantarius
Streptococcus	gallolyticus	0.1765	Streptococcus_gallolyticus_alactolyticus
Streptococcus	alactolyticus	0.0552	Streptococcus_gallolyticus_alactolyticus
Streptococcus	bovis	0.011	Streptococcus_gallolyticus_alactolyticus
Streptococcus	gordonii	0.0993	Streptococcus_gordonii
Streptococcus	intermedius	0.0441	Streptococcus_intermedius
Streptococcus	mitis_oralis	4.1363	Streptococcus_mitis
Streptococcus	parasanguinis	0.353	Streptococcus_parasanguinis
Streptococcus	pluranimalium	1.2133	Streptococcus_pluranimalium
Streptococcus	pneumoniae	1.9082	Streptococcus_pneumoniae
Streptococcus	porcinus	0.011	Streptococcus_porcinus
Streptococcus	pyogenes	0.0882	Streptococcus_pyogenes
Streptococcus	salivarius	1.5111	Streptococcus_salivarius_vestibularis
Streptococcus	vestibularis	0.2096	Streptococcus_salivarius_vestibularis
Streptococcus	thermophilus	0.0221	Streptococcus_salivarius_vestibularis
Streptococcus	sanguinis	0.8273	Streptococcus_sanguinis
Streptococcus	suis_ii	0.0331	Streptococcus_suis
Streptococcus	thoraltensis	0.0221	Streptococcus_thoraltensis
Streptococcus	uberis	0.0552	Streptococcus_uberis

Yersinia	enterocolitica	0.011	Yersinia_enterocolitica

Supplementary Table 5: Cultured species not included in the reference database, the percentage of counts they represent from their genus and all samples.

**Species**	**Percent**
Staphylococcus_GroupA	27.9156
Escherichia/Shigella_coli_sonnei	15.2391
Enterobacteriaceae_GroupA	9.2504
Enterococcus_faecalis_durans_hirae	6.304
Moraxella_catarrhalis_nonliquefaciens	5.5913
Streptococcus_mitis	5.1391
Micrococcus_luteus	2.3845
Streptococcus_pneumoniae	2.3708
Clostridium_difficile	2.1105
Streptococcus_salivarius_vestibularis	2.0419
Enterococcus_raffinosus_avium	1.809
Haemophilus_influenzae	1.5075
Streptococcus_pluranimalium	1.5075
Streptococcus_agal_dys	1.3978
Enterococcus_casseliflavus	1.1512
Streptococcus_sanguinis	1.0278
Rothia_mucilaginosa	0.8771
Staphylococcus_haemolyticus	0.7263
Gemella_GroupA	0.6989
Enterococcus_faecium	0.5893
Staphylococcus_intermedius	0.5071
Micrococcus_lylae	0.4934
Proteus_mirabilis	0.4522
Kocuria_kristinae	0.4385
Streptococcus_parasanguinis	0.4385
Sphingomonas_paucimobilis	0.4248
Stenotrophomonas_maltophilia	0.4248
Acinetobacter_lwoffii	0.37
Streptococcus_dysgalactiae	0.3426
Granulicatella_adiacens	0.3152
Haemophilus_parainfluenzae	0.3015
Streptococcus_gallolyticus_alactolyticus	0.3015
Citrobacter_koseri	0.2878
Kocuria_rosea	0.2741
Enterobacter_aerogenes	0.2604
Escherichia_hermannii	0.2193
Staphylococcus_lentus	0.2193
Serratia_marcescens	0.2056

Streptococcus_equinus_infantarius	0.2056
Streptococcus_constellatus	0.1782
Neisseria_cinerea_subflava_perflava	0.1507
Raoultella_planticola	0.1507
Proteus_penneri	0.137
Dermacoccus_nishinomiyaensis	0.1233
Neisseria_sicca_mucosa	0.1233
Proteus_vulgaris	0.1233
Staphylococcus_carnosus_simulans	0.1233
Streptococcus_gordonii	0.1233
Erysipelothrix_rhusiopathiae	0.1096
Granulicatella_elegans	0.1096
Neisseria_flavescens	0.1096
Pseudomonas_aeruginosa	0.1096
Streptococcus_pyogenes	0.1096
Staphylococcus_cohnii	0.0959
Streptococcus_anginosus	0.0959
Aeromonas_caviae_salmonicida_veronii	0.0822
Lactococcus_lactis	0.0822
Morganella_morganii	0.0822
Serratia_liquefaciens	0.0822
Lactococcus_garvieae	0.0685
Raoultella_ornithinolytica	0.0685
Serratia_fonticola	0.0685
Staphylococcus_auricularis	0.0685
Staphylococcus_saprophyticus	0.0685
Streptococcus_uberis	0.0685
Cronobacter_sakazakii	0.0548
Hafnia_alvei	0.0548
Lactococcus_raffinolactis	0.0548
Pediococcus_pentosaceus	0.0548
Streptococcus_intermedius	0.0548
Acinetobacter_baumannii	0.0411
Acinetobacter_ursingii	0.0411
Aerococcus_viridans	0.0411
Enterobacter_amnigenus	0.0411
Neisseria_elongata	0.0411
Pasteurella_canis	0.0411
Pseudomonas_oryzihabitans	0.0411
Staphylococcus_xylosus	0.0411
Streptococcus_suis	0.0411
Acinetobacter_junii	0.0274
Alloiococcus_otitis	0.0274

Clostridium_perfringens	0.0274
Enterobacter_aburiae	0.0274
Gardnerella_vaginalis	0.0274
Kocuria_varians	0.0274
Providencia_rettgeri	0.0274
Pseudomonas_fluorescens	0.0274
Pseudomonas_luteola	0.0274
Staphylococcus_chromogenes	0.0274
Streptococcus_thoraltensis	0.0274
Acinetobacter_haemolyticus	0.0137
Aeromonas_sobria	0.0137
Enterococcus_columbae	0.0137
Haemophilus_haemolyticus	0.0137
Neisseria_meningitidis	0.0137
Oligella_ureolytica	0.0137
Oligella_urethralis	0.0137
Pantoea_agglomerans	0.0137
Pasteurella_pneumotropica	0.0137
Providencia_alcalifaciens	0.0137
Pseudomonas_stutzeri	0.0137
Staphylococcus_coctunansis	0.0137
Staphylococcus_sciuri	0.0137
Staphylococcus_vitulinus	0.0137
Streptococcus_porcinus	0.0137
Yersinia_enterocolitica	0.0137

Supplementary Table 6: Bacteria identified by culturing, amplicon sequencing or both. The counts represent the number of bacteria identified per type of sample. [+]calculated as both/(both+cultured), *calculated as sequenced/(both+sequenced). Groups were divided into dominant (>10%), major (1-10%), or minor (<1%) components of each sample based upon their relative abundance within each sample.

**Area**	**Group**	**Cultured Only**	**Sequenced Only**	**Both**	**Culturing found by sequencing**	**Sequencing found by culturing**
All	All	1608	15978	5008	75.70%	23.86%
	Dominant		3282	3251		49.76%
	Major		5221	1137		17.88%
	Minor		7475	620		7.66%
Fecal	All	899	6586	2342	72.26%	26.23%
	Dominant		1489	1559		51.15%
	Major		2360	534		18.45%
	Minor		2737	249		8.34%
Hypopharyngeal	All	709	9392	2666	78.99%	22.11%
	Dominant		1793	1692		48.55%
	Major		2861	603		17.41%
	Minor		4738	371		7.26%

Supplementary Table 7: Comparison of the 106 unique bacteria identified by amplicon sequencing and culture methods in the 3,538 samples. *Bacterial genera have more than one species which have identical V4 region. #Amplicon sequencing were divided into dominant (>10%), major (1-10%), or minor (<1%) components of each sample based upon their relative abundance within each sample. No sequence means that we didn't observe any hit within each sample. $Culturing were divided into absent which represent the number of negative samples and present which represent a number of positive samples.

**Amplicon sequencing#**	**No Sequence**		**Minor**		**Major**		**Dominant**	
**Culturing$**	**Absent**	**Present**	**Absent**	**Present**	**Absent**	**Present**	**Absent**	**Present**
Acinetobacter_baumannii	3400	3	100	0	21	0	14	0
Acinetobacter_haemolyticus	3526	1	6	0	4	0	1	0
Acinetobacter_junii	3453	1	61	0	17	0	5	1
Acinetobacter_lwoffii	3425	23	55	0	27	2	4	2
Acinetobacter_ursingii	3523	3	9	0	3	0	0	0
Aerococcus_viridans	3530	3	4	0	1	0	0	0
Aeromonas_caviae_salmonicida_veronii*	3527	5	5	1	0	0	0	0
Aeromonas_sobria	3537	1	0	0	0	0	0	0
Alloiococcus_otitis	3535	2	0	0	1	0	0	0
Citrobacter_koseri	3487	8	10	1	10	3	10	9
Clostridium_difficile	3247	149	43	3	71	1	23	1
Clostridium_perfringens	3005	1	210	0	213	0	108	1
Cronobacter_sakazakii	3533	4	1	0	0	0	0	0
Dermacoccus_nishinomiyaensis	3526	9	3	0	0	0	0	0
Enterobacter_aburiae	3536	2	0	0	0	0	0	0
Enterobacter_aerogenes	3519	19	0	0	0	0	0	0
Enterobacter_amnigenus	3535	3	0	0	0	0	0	0
Enterobacteriaceae_GroupA*	2102	189	403	47	212	106	146	333
Enterococcus_casseliflavus	3454	84	0	0	0	0	0	0
Enterococcus_columbae	3536	0	1	0	0	1	0	0
Enterococcus_faecalis_durans_hirae*	2646	173	245	95	160	113	27	79
Enterococcus_faecium	3495	43	0	0	0	0	0	0
Enterococcus_raffinosus_avium*	3370	106	23	3	10	18	3	5
Erysipelothrix_rhusiopathiae	3530	8	0	0	0	0	0	0
Escherichia/Shigella_coli_sonnei*	1439	65	508	37	243	110	236	900
Escherichia_hermannii	3522	16	0	0	0	0	0	0
Gardnerella_vaginalis	3536	2	0	0	0	0	0	0
Gemella_GroupA*	1939	4	670	5	646	22	232	20
Granulicatella_adiacens	3515	23	0	0	0	0	0	0
Granulicatella_elegans	3206	7	206	1	107	0	11	0

Haemophilus_haemolyticus	2306	0	650	0	372	0	209	1
Haemophilus_influenzae	3428	110	0	0	0	0	0	0
Haemophilus_parainfluenzae	1853	3	606	3	542	10	515	6
Hafnia_alvei	3534	4	0	0	0	0	0	0
Kocuria_kristinae	3505	32	1	0	0	0	0	0
Kocuria_rosea	3518	20	0	0	0	0	0	0
Kocuria_varians	3536	2	0	0	0	0	0	0
Lactococcus_garvieae	3533	5	0	0	0	0	0	0
Lactococcus_lactis	3486	6	36	0	6	0	4	0
Lactococcus_raffinolactis	3534	4	0	0	0	0	0	0
Micrococcus_luteus	3203	159	122	12	31	3	8	0
Micrococcus_lylae	3498	36	2	0	2	0	0	0
Moraxella_catarrhalis_nonliquefaciens*	1925	7	685	18	302	44	218	339
Morganella_morganii	3513	3	11	2	5	1	3	0
Neisseria_cinerea_subflava_perflava*	2828	2	394	1	202	3	103	5
Neisseria_elongata	3531	3	3	0	1	0	0	0
Neisseria_flavescens	3530	8	0	0	0	0	0	0
Neisseria_meningitidis	3395	0	93	0	33	0	16	1
Neisseria_sicca_mucosa	3513	9	9	0	5	0	2	0
Oligella_ureolytica	3537	1	0	0	0	0	0	0
Oligella_urethralis	3536	1	0	0	0	0	1	0
Pantoea_agglomerans	3527	0	6	0	3	0	1	1
Pasteurella_canis	3534	3	1	0	0	0	0	0
Pasteurella_pneumotropica	3537	1	0	0	0	0	0	0
Pediococcus_pentosaceus	3530	4	1	0	2	0	1	0
Proteus_mirabilis	3505	33	0	0	0	0	0	0
Proteus_penneri	3528	10	0	0	0	0	0	0
Proteus_vulgaris	3508	8	12	0	8	0	1	1
Providencia_alcalifaciens	3533	0	4	0	0	0	0	1
Providencia_rettgeri	3536	2	0	0	0	0	0	0
Pseudomonas_aeruginosa	3427	6	74	0	20	2	9	0
Pseudomonas_fluorescens	3536	2	0	0	0	0	0	0
Pseudomonas_luteola	3535	2	1	0	0	0	0	0
Pseudomonas_oryzihabitans	3535	3	0	0	0	0	0	0
Pseudomonas_stutzeri	3534	1	2	0	1	0	0	0
Raoultella_ornithinolytica	3513	5	9	0	6	0	5	0
Raoultella_planticola	3504	8	12	0	8	0	3	3
Rothia_mucilaginosa	3470	64	2	0	2	0	0	0
Serratia_fonticola	3527	5	4	0	1	0	1	0
Serratia_liquefaciens	3532	6	0	0	0	0	0	0
Serratia_marcescens	3511	10	8	0	2	0	2	5
Sphingomonas_paucimobilis	3506	31	0	0	0	0	1	0
Staphylococcus_auricularis	3468	4	31	1	25	0	9	0

Staphylococcus_carnosus_simulans*	3526	9	1	0	2	0	0	0
Staphylococcus_chromogenes	3532	2	4	0	0	0	0	0
Staphylococcus_coctunansis	3537	1	0	0	0	0	0	0
Staphylococcus_cohnii	3531	7	0	0	0	0	0	0
Staphylococcus_GroupA	754	130	425	319	236	497	86	1091
Staphylococcus_haemolyticus	3485	53	0	0	0	0	0	0
Staphylococcus_intermedius	3482	32	18	0	0	2	1	3
Staphylococcus_lentus	3522	16	0	0	0	0	0	0
Staphylococcus_saprophyticus	3533	5	0	0	0	0	0	0
Staphylococcus_sciuri	3536	1	1	0	0	0	0	0
Staphylococcus_vitulinus	3537	1	0	0	0	0	0	0
Staphylococcus_xylosus	3535	3	0	0	0	0	0	0
Stenotrophomonas_maltophilia	3500	29	6	2	1	0	0	0
Streptococcus_agal_dys	3255	29	121	14	43	42	17	17
Streptococcus_anginosus	3409	6	62	0	39	1	21	0
Streptococcus_constellatus	3519	13	4	0	1	0	1	0
Streptococcus_dysgalactiae	3513	25	0	0	0	0	0	0
Streptococcus_equinus_infantarius*	3486	6	13	0	14	4	10	5
Streptococcus_gallolyticus_alactolyticus*	3489	16	8	1	14	2	5	3
Streptococcus_gordonii	3529	9	0	0	0	0	0	0
Streptococcus_intermedius	3524	4	7	0	2	0	1	0
Streptococcus_mitis	1382	16	527	17	555	46	699	296
Streptococcus_parasanguinis	3506	32	0	0	0	0	0	0
Streptococcus_pluranimalium	3428	110	0	0	0	0	0	0
Streptococcus_pneumoniae	2959	51	143	9	164	30	99	83
Streptococcus_porcinus	3537	1	0	0	0	0	0	0
Streptococcus_pyogenes	3512	0	18	1	0	5	0	2
Streptococcus_salivarius_vestibularis*	1392	16	774	27	827	77	396	29
Streptococcus_sanguinis	3455	75	4	0	4	0	0	0
Streptococcus_suis	3535	3	0	0	0	0	0	0
Streptococcus_thoraltensis	3536	2	0	0	0	0	0	0
Streptococcus_uberis	3528	5	2	0	3	0	0	0
Yersinia_enterocolitica	3537	1	0	0	0	0	0	0
