# Supplementary material for: Amplicon sequencing provides more accurate microbiome information in healthy children compared to culturing
Source: Commun Biol. 2019 Aug 5;2:291. doi: 10.1038/s42003-019-0540-1 (PMC6683184; doi:10.1038/s42003-019-0540-1)
Supplement: Supplementary file 2 — Description of Additional Supplementary Files [file 42003_2019_540_MOESM2_ESM.docx]

**Description of Additional Supplementary Files**

**Supplementary Data 1**

The contingency table showing which bacteria were isolated from each sample.
