# Supplementary material for: Amplicon sequencing provides more accurate microbiome information in healthy children compared to culturing
Source: Commun Biol. 2019 Aug 5;2:291. doi: 10.1038/s42003-019-0540-1 (PMC6683184; doi:10.1038/s42003-019-0540-1)
Supplement: Supplementary file 4 — Reporting Summary [file 42003_2019_540_MOESM4_ESM.pdf]

nature research

Corresponding author(s): Søren Johannes Sørensen
Karen A. Krogfelt

Last updated by author(s): Mar 14, 2019

Reporting Summary

Nature Research wishes to improve the reproducibility of the work that we publish. This form provides structure for consistency and transparency in reporting. For further information on Nature Research policies, see Authors & Referees and the Editorial Policy Checklist.

Statistics

For all statistical analyses, confirm that the following items are present in the figure legend, table legend, main text, or Methods section.

n/a	Confirmed	
☐	☒	The exact sample size (*n*) for each experimental group/condition, given as a discrete number and unit of measurement
☐	☒	A statement on whether measurements were taken from distinct samples or whether the same sample was measured repeatedly
☒	☐	The statistical test(s) used AND whether they are one- or two-sided<br>*Only common tests should be described solely by name; describe more complex techniques in the Methods section.*
☒	☐	A description of all covariates tested
☒	☐	A description of any assumptions or corrections, such as tests of normality and adjustment for multiple comparisons
☐	☒	A full description of the statistical parameters including central tendency (e.g. means) or other basic estimates (e.g. regression coefficient) AND variation (e.g. standard deviation) or associated estimates of uncertainty (e.g. confidence intervals)
☐	☒	For null hypothesis testing, the test statistic (e.g. *F*, *t*, *r*) with confidence intervals, effect sizes, degrees of freedom and *P* value noted<br>*Give P values as exact values whenever suitable.*
☒	☐	For Bayesian analysis, information on the choice of priors and Markov chain Monte Carlo settings
☒	☐	For hierarchical and complex designs, identification of the appropriate level for tests and full reporting of outcomes
☒	☐	Estimates of effect sizes (e.g. Cohen's *d*, Pearson's *r*), indicating how they were calculated

Our web collection on statistics for biologists contains articles on many of the points above.

Software and code

Policy information about availability of computer code

Data collection	No software was used to collect the data
Data analysis	cutadapt (v1.15), QIIME2 (v 2018.2), QIIME (v 1.9.1), R (v 3.5.0). We described the usage of each software in details in the METHODS.

For manuscripts utilizing custom algorithms or software that are central to the research but not yet described in published literature, software must be made available to editors/reviewers. We strongly encourage code deposition in a community repository (e.g. GitHub). See the Nature Research guidelines for submitting code & software for further information.

Data

Policy information about availability of data

All manuscripts must include a data availability statement. This statement should provide the following information, where applicable:

- Accession codes, unique identifiers, or web links for publicly available datasets
- A list of figures that have associated raw data
- A description of any restrictions on data availability

The sequences were submitted in NCBI Sequence Read Archive (SRA) under the Bioproject ID PRJNA543007.

Field-specific reporting

Please select the one below that is the best fit for your research. If you are not sure, read the appropriate sections before making your selection.

☒ Life sciences ☐ Behavioural & social sciences ☐ Ecological, evolutionary & environmental sciences

For a reference copy of the document with all sections, see nature.com/documents/nr-reporting-summary-flat.pdf

Life sciences study design

All studies must disclose on these points even when the disclosure is negative.

Sample size	Hypopharyngeal aspirates were collected at 1 week (537 samples), 1 month (626 samples), and 3 months (627samples) after birth. Fecal samples were collected at 1 week (542 samples), 1 month (597 samples), and 12 months (609 samples)
Data exclusions	Exclusion criteria were chronic cardiac, endocrinological, nephrological or lung disease other than asthma.
Replication	The study has not been replicated as we do not have access to a similar sample collection nor the funding or time to perform a replication of this study. Furthermore, the DNA extraction from hypopharyngeal samples used the entirety of the samples and cannot be reproduced.
Randomization	Not applicable.
Blinding	Not applicable.

Reporting for specific materials, systems and methods

We require information from authors about some types of materials, experimental systems and methods used in many studies. Here, indicate whether each material, system or method listed is relevant to your study. If you are not sure if a list item applies to your research, read the appropriate section before selecting a response.

Materials & experimental systems

n/a	Involved in the study	
☒	☐	Antibodies
☒	☐	Eukaryotic cell lines
☒	☐	Palaeontology
☒	☐	Animals and other organisms
☐	☒	Human research participants
☒	☐	Clinical data

Methods

n/a	Involved in the study	
☒	☐	ChIP-seq
☒	☐	Flow cytometry
☒	☐	MRI-based neuroimaging

Human research participants

Policy information about studies involving human research participants

Population characteristics	The Copenhagen Prospective Studies on Asthma in Childhood 2010 (COPSAC2010) cohort is a population-based Danish mother-child cohort of 738 women and their 700 children recruited at week 24 of pregnancy. The children have been followed prospectively from birth at the COPSAC research unit with deep clinical phenotyping with scheduled visits during the first 8 years of life. The study pediatricians collected all information during these clinical visits scheduled at 1 week, 1, 3, 6, 12, 18, 24, 30, and 36 months, and yearly thereafter. Additional acute care visits were arranged whenever the children experienced lung or skin symptoms. For the current study we are only using data from the first year of their lives and not any clinical phenotypes.
Recruitment	A total of 738 pregnant women were prospectively enrolled in pregnancy week 24. Exclusion criteria were daily intake of more than 600 IU of Vitamin D and having known endocrine (e.g. diabetes or thyroid disease), kidney, or heart diseases. The study was approved by all relevant authorities and all women provided informed consent prior to participation.
Ethics oversight	Danish Ethical Committee (H-B-2008-093), the Danish Data Protection Agency (2015-41-3696)

Note that full information on the approval of the study protocol must also be provided in the manuscript.
